# Debunking the idea of biological optimisation: quantitative biology to the rescue

**DOI:** 10.1017/qpb.2024.3

**Published:** 2024-04-03

**Authors:** Olivier Hamant

**Affiliations:** Laboratoire de Reproduction et Développement des Plantes, Université de Lyon, ENS de Lyon, UCBL, INRAE, CNRS, INRIA 46 Allée d’Italie, Lyon, France

**Keywords:** optimisation, plant science, quantitative biology, robustness, systems biology

## Abstract

The idea that plants would be efficient, frugal or optimised echoes the recurrent semantics of ‘blueprint’ and ‘program’ in molecular genetics. However, when analysing plants with quantitative approaches and systems thinking, we instead find that plants are the results of stochastic processes with many inefficiencies, incoherence or delays fuelling their robustness. If one had to highlight the main value of quantitative biology, this could be it: plants are robust systems because they are not efficient. Such systemic insights extend to the way we conduct plant research and opens plant science publication to a much broader framework.

When you open any book in biology (e.g., Alberts, 2022), you find a number of diagrams recapitulating molecular cascades or dissecting the components of macromolecular complexes. While this iconography is very useful to get a global picture of the different elements involved in certain responses (e.g., the photosynthetic antenna or the abscisic acid signalling pathway), it tells us very little about the dynamics, and thus the behaviour, of the corresponding mechanisms. In fact, it may in contrast reinforce the idea that a plant is the sum of its parts, a bit like a machine (Nicholson, 2019). Why is this a problem?

In short, this contributes to the false idea that plants, like all living beings, would be optimised during evolution. Paradoxically, while the authors in these books rightfully condemn creationism, they often use an iconography that is consistent with intelligent design. The semantics also fuels this discourse: ‘master genes’, ‘blueprint’, ‘genetic program’, and so forth.

This paradox may have started when we had a biased reading of *On the origin of species* (Darwin, 1859). In Chapter 1 of his book, Darwin rooted the theory of natural selection in artificial selection, that is, domestication. This is not a coincidence: with agriculture becoming more and more rationalised, Darwin was inspired by the acceleration of domestication, with observable macroscopic phenotypic modifications within his lifetime. Furthermore, the increase in resolution, notably with microscopy, allowed the detection of smaller changes. While evolution occurs in both artificial and natural selection, the ‘goals’ are different. In the case of man-made domestication, there is a drive towards optimising agriculture, plant yields and/or resistance to pathogens. In contrast, there is no goal in the case of natural selection: evolution is blind and new characters are the emerging properties of contingencies. In other words, artificial selection is the result of a top-down approach (a rather reductionistic angle), whereas natural selection is the result of a bottom-up approach (a rather systemic angle).

One could say that this is not a major problem since the molecular mechanisms are the same: in the end, this all comes down to genetic heredity. It remains that such a bias on optimisation is very problematic. This could indeed lead to the idea that evolution, whether under natural or artificial selection, is an optimisation force. In fact, this idea is prevalent in biomimicry: we would copy nature because it would have optimised certain processes. For instance, Velcro was invented when Swiss electrical engineer George de Mestral observed how burdock seeds could cling on his coat. Another famous biomimicry example is the case of superhydrophobicity of lotus surfaces thanks to nano- and micrometric wax textures (Vonna, 2023). However, biomimicry is the wrong name for a nice idea: we are not inspired by nature; we rather apply our technical needs on natural artefacts (Figure 1). Burdock and lotus may have wonderful seed dispersal and self-cleaning strategies, but, as global systems, their photosynthesis yields are still under 2% (like all plants), meaning that they waste more than 98% of solar energy to produce their remarkable seeds and leaves (Arp et al., 2020). This does not fit the optimisation narrative.

**Figure 1.**
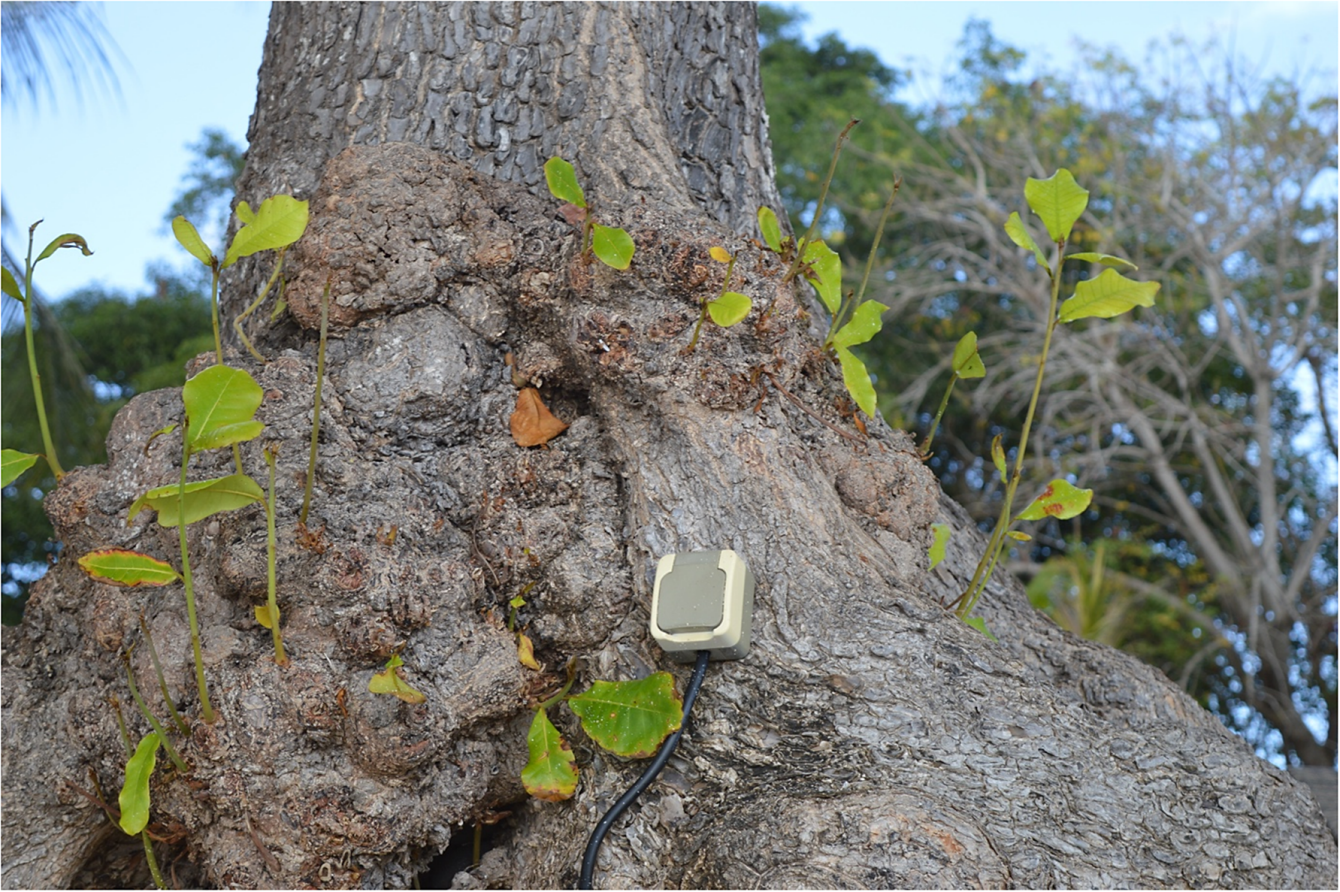
Reductionistic versus systemic views on plants: Should we use plants to feed our needs, or should we use plant lessons to question our choices? *Source:* Author’s own.

To get clarity, one could follow Dwight Eisenhower’s famous quote: ‘If you can’t solve a problem, enlarge it’. This is a call for systems thinking. Thanks to quantitative approaches, we now find that many biological processes are random (feeding the subfield of stochastic biology; see, e.g., Giannakis et al., 2022; Saltini & Mulder, 2021), incoherent (see, e.g., the many incoherent feedforward loops in all networks; Kuan et al., 2022), redundant (from multigenic families (Samalova et al., 2022) to leaf number on trees), heterogeneous (see, e.g., how organs reach reproducible shapes despite having variable tissue topologies; Roeder, 2021), slow (see, e.g., how oscillatory behaviours rely on the active maintenance of delays; Shimadzu et al., 2022) and inefficient (see, e.g., the energy it takes to maintain the electric potential of plasma membranes; Rubio et al., 2020). The more we analyse plants, the more we find that they are not optimised. Instead, they contain and fuel many inefficiencies to remain adaptable. Thus, they are neither perfectly adapted, nor optimised for high performance.

This has been theorised, with the idea that evolution is not a route towards increased performance (a very anthropomorphic idea of progress), but is instead, and more pragmatically, the product of stress tests. What is selected during evolution is fitness. And, systems biology like systems engineering or cybernetics is crystal clear: high robustness is incompatible with high efficiency. In a more generic way, one could say that plants are very robust, because they are not very efficient. This can be demonstrated at all scales. Ulanowicz et al. (2009) identified the trade-off between robustness and efficiency by studying ecosystems. Niklas (1994) also reached the same conclusion when analysing the biophysics of living systems.

Interestingly, a necessary key to this demonstration lies in the quantitative approach. When analysing the interactions and emerging properties, we can derive theories that are less biased by human perception (here, the idea that evolution would be an optimisation force), we can formalise the dynamics of systems and we can open novel and exciting questions in plant science (e.g., the role of stochasticity in plant development or the shielding role of incoherent feedforward loops against external perturbations) (Autran et al., 2021).

To conclude, if we had to consider biomimicry literally, that is, as an inspiration from living systems for our societies, we should then favour robustness instead of efficiency. It would be the exact opposite of valuing optimisation. Needless to say, in a world experiencing increasing socio-ecological and geopolitical turbulence, this lesson from quantitative biology is very apt. This might in fact be the main, overarching, lesson from quantitative biology, in all kingdoms (Hamant, 2022).

At *Quantitative Plant Biology*, we take such an integrative and dynamic approach very seriously, precisely because this is a way to enforce scientific rigour, while also opening the plant science field to a much wider array of questions and partners (Autran et al., 2021). Systems thinking naturally incites us to broaden the scope of interaction within and outside the plant science community, not only to reach a more integrative view on plants, but also to conduct stress tests on our results. This notably includes multiscale biology, citizen science or art and science. In other words, quantitative plant biology is providing robust scientific results, because it is rooted in creative, interdisciplinary and intercultural approaches. This community is rapidly growing, and is more and more pertinent as science and society become increasingly divided. *Quantitative Plant Biology* is a forum to welcome your contributions to this global effort.

## Data Availability

No data are shown in this article.
